# The Diagnostic Role of Shear Wave Elastography and Renal Resistive Index in Diabetic Patients With and Without Chronic Kidney Disease: A Cross-Sectional Observational Study

**DOI:** 10.7759/cureus.110767

**Published:** 2026-06-13

**Authors:** Muskan Bansal, Amit Jain, Sudhir Mehta

**Affiliations:** 1 Department of Radiodiagnosis, Maharishi Markandeshwar Institute of Medical Sciences and Research, Mullana, IND; 2 Department of Nephrology, Maharishi Markandeshwar Institute of Medical Sciences and Research, Mullana, IND

**Keywords:** chronic kidney disease, diabetes mellitus, diabetic kidney disease, renal resistive index, shear wave elastography

## Abstract

Background and objective

Diabetic kidney disease (DKD) is among the most common and serious complications of diabetes mellitus and is a major cause of chronic kidney disease (CKD) worldwide. Conventional biochemical markers such as serum creatinine, estimated glomerular filtration rate (eGFR), and albumin-creatinine ratio (ACR) primarily reflect functional impairment and may fail to detect early structural renal changes. Shear wave elastography (SWE) and renal resistive index (RRI) are emerging non-invasive imaging modalities that may help in the early identification of renal fibrosis and vascular alterations in diabetic nephropathy. The present study aimed to evaluate and compare SWE and RRI findings in diabetic patients with and without CKD and to assess their correlation with conventional renal function parameters.

Methods

This cross-sectional analytical observational study was conducted in the Department of Radiodiagnosis at Maharishi Markandeshwar Institute of Medical Sciences and Research, Mullana, Ambala, from April 2025 to March 2026. A total of 150 diabetic patients were enrolled, including 75 patients with clinically proven CKD and 75 diabetic patients without CKD who served as controls. Clinical and laboratory parameters, including serum creatinine, eGFR, ACR, and BMI, were recorded. Ultrasonography, Doppler evaluation, and SWE of both kidneys were performed using a Philips EPIQ-7 GI ultrasound machine (Philips Healthcare, Best, the Netherlands). Mean SWE values were recorded in kilopascals (kPa), while RRI was assessed using Doppler ultrasonography.

Results

A total of 150 diabetic patients were enrolled, including 75 with CKD and 75 without CKD. The groups were comparable in age (59.19 ± 7.95 vs. 58.80 ± 8.58 years; p = 0.775), sex distribution (49 (65.3%) males vs. 45 (60.0%) males; p = 0.613), and BMI (24.36 ± 3.03 vs. 23.58 ± 3.21 kg/m²; p = 0.130). Patients with CKD had significantly lower eGFR (59.92 ± 36.08 vs. 111.52 ± 12.72 ml/min/1.73 m²; p < 0.001) and higher serum creatinine (2.63 ± 1.37 vs. 1.36 ± 0.46 mg/dl), ACR (201.17 ± 214.51 vs. 11.57 ± 7.57 mg/g), RRI (0.69 ± 0.07 vs. 0.51 ± 0.10), and SWE values (11.19 ± 2.82 vs. 8.31 ± 0.82 kPa) (all p < 0.001). Among CKD patients, 20 (26.7%) were in Stage 1 and 20 (26.7%) in Stage 2, 10 (13.3%) in Stage 3, 15 (20.0%) in Stage 4, and 10 (13.3%) in Stage 5. SWE showed a strong negative correlation with eGFR (r = -0.777) and a positive correlation with ACR (r = 0.791) (both p < 0.001). RRI demonstrated an area under the curve (AUC) of 0.924 with 72% sensitivity and 100% specificity, while SWE showed an AUC of 0.899 with 100% sensitivity and 68% specificity for predicting CKD.

Conclusions

SWE and RRI are valuable non-invasive imaging biomarkers for the assessment of DKD. Both parameters showed a significant association with renal dysfunction and CKD severity. SWE demonstrated high sensitivity, while RRI demonstrated high specificity for identifying CKD. These modalities may serve as useful non-invasive adjuncts for assessment and risk stratification in diabetic patients. Further longitudinal studies are required to establish their role in early detection, monitoring, and prognostic evaluation.

## Introduction

Diabetes mellitus is one of the major global health challenges, with a steadily rising prevalence and increasing burden on healthcare systems worldwide. Apart from chronic hyperglycemia, diabetes causes progressive structural and functional damage to multiple organs. Diabetic kidney disease (DKD) is among the most serious complications and is a leading cause of chronic kidney disease (CKD) and end-stage kidney disease (ESKD). Persistent hyperglycemia leads to microvascular injury, oxidative stress, inflammation, and renal fibrosis, resulting in progressive kidney damage. Early detection is important because timely intervention can slow disease progression [1].

Routine evaluation of DKD includes serum creatinine, blood urea nitrogen, urinary albumin excretion, and estimated glomerular filtration rate (eGFR). Persistent albuminuria with reduced eGFR strongly suggests DKD. However, these markers mainly reflect functional impairment rather than early structural changes, so significant renal damage may already be present before abnormalities become clinically evident [2]. Ultrasonography is commonly used because it is non-invasive, inexpensive, and widely available. In advanced disease, kidneys may show increased echogenicity and reduced cortical thickness, but conventional ultrasonography has limited sensitivity for detecting early diabetic renal injury [3]. Renal biopsy remains the gold standard for assessing renal fibrosis, but it is invasive and associated with risks such as bleeding, infection, pain, and sampling error [4]. Therefore, there is increasing interest in non-invasive techniques for evaluating renal fibrosis and disease progression.

Shear wave elastography (SWE) is a non-invasive ultrasound-based imaging technique that measures tissue stiffness by assessing the velocity of shear waves through tissue. Stiffer tissues transmit waves faster, making SWE a potential indirect marker of fibrosis [5]. Initially used in liver disease assessment, SWE is now increasingly applied in renal diseases because it is radiation-free, cost-effective, widely available, and suitable for repeated monitoring [2]. Several studies have reported differences in renal cortical stiffness between healthy individuals and patients with CKD. In DKD, SWE values have shown correlations with albuminuria, decline in eGFR, and Doppler abnormalities, suggesting its role as an early marker of renal structural damage [1]. Some studies also indicate that diabetic patients without overt biochemical abnormalities may still show altered renal stiffness compared to healthy controls, supporting the possibility of detecting subclinical disease using SWE [6].

However, findings across studies remain inconsistent because renal stiffness is influenced not only by fibrosis but also by perfusion, inflammation, edema, and technical factors such as hydration status, region-of-interest placement, and operator experience [7]. Variations in study protocols and SWE devices have also contributed to conflicting results. Recent advances, such as SWE-based radiomics and machine learning models, have shown promise in predicting renal and cardiovascular outcomes in diabetic patients [8]. Despite growing interest, few studies have directly compared SWE findings between diabetic patients with CKD and those without CKD using standardized methods [9].

Therefore, evaluating SWE in diabetic patients with and without CKD may help clarify its diagnostic value and association with renal dysfunction. SWE may serve as a useful non-invasive tool for assessment and risk stratification in DKD. The primary objective of this study was to evaluate and compare SWE and renal resistive index (RRI) in diabetic patients with and without CKD. The secondary objective was to assess the correlation of these imaging biomarkers with conventional renal function parameters, including eGFR and albumin-creatinine ratio (ACR). By simultaneously evaluating SWE and RRI in diabetic patients across different stages of renal involvement, this study aims to provide additional evidence regarding their potential role as non-invasive imaging biomarkers in DKD.

## Materials and methods

Study design and setting

This cross-sectional analytical observational study was conducted in the Department of Radiodiagnosis, Maharishi Markandeshwar Institute of Medical Sciences and Research, Mullana, Ambala, after obtaining approval from the Institutional Scientific and Ethical Committee Review Board (Letter No. IEC-3414). The study was carried out from April 2025 to March 2026, including both data collection and thesis preparation.

Study population

The study included adult diabetic patients undergoing ultrasonography in the Department of Radiodiagnosis. A total of 150 participants were enrolled: 75 diabetic patients with clinically proven CKD and 75 diabetic patients without CKD who served as controls. The sample size was calculated with an alpha error of 0.05 and a study power of 90%, based on comparison of SWE values between the two groups. Eligible participants were recruited consecutively until the desired sample size was achieved. CKD was diagnosed according to established clinical criteria based on persistent kidney damage and/or reduced kidney function, including eGFR and ACR findings documented in the patients' medical records. Patients with CKD Stages 1 and 2 were included based on evidence of kidney damage despite preserved or near-preserved eGFR.CKD staging was performed according to the Kidney Disease: Improving Global Outcomes (KDIGO) 2024 Clinical Practice Guideline. CKD Stage 1 was defined as eGFR ≥ 90 mL/min/1.73 m² with evidence of kidney damage; Stage 2 as eGFR 60-89 mL/min/1.73 m² with evidence of kidney damage; Stage 3 as eGFR 30-59 mL/min/1.73 m²; Stage 4 as eGFR 15-29 mL/min/1.73 m²; and Stage 5 as eGFR < 15 mL/min/1.73 m² [10].

Inclusion and exclusion criteria

Patients aged 18 to 75 years with diabetes mellitus were included in the study. Both diabetic patients with clinically diagnosed CKD and diabetic patients without CKD were enrolled. Patients with morbid obesity (BMI > 40 kg/m²), respiratory problems, renal artery stenosis, a history of renal transplant, or those on maintenance hemodialysis were excluded from the study.

Patient evaluation and data collection

After screening according to the inclusion and exclusion criteria, eligible participants were informed about the study in detail. Written informed consent was obtained from all participants before enrollment. For illiterate participants, thumb impressions along with witness signatures were taken. Clinical history and examination findings were recorded for each patient. Relevant laboratory and clinical parameters, including serum creatinine, eGFR, ACR, BMI, and CKD stage, were documented.

Ultrasonography and SWE technique

Ultrasonography and SWE of both kidneys were performed using a Philips EPIQ-7 GI ultrasound machine (Philips Healthcare, Best, the Netherlands). Patients were advised to fast for at least four to six hours before the examination and were instructed to empty their bladder before the procedure. Examinations were carried out in supine, prone, or lateral decubitus positions depending on image quality. Imaging was performed during maximum inspiration to reduce kidney movement and obtain better visualization. All SWE measurements were obtained during breath-hold at maximum inspiration to minimize respiratory motion artifacts, and minimal transducer pressure was applied throughout the examination to reduce measurement variability.

The transducer was placed longitudinally over the kidneys. Gray-scale settings were optimized for adequate brightness, contrast, and spatial resolution. A 10 mm × 10 mm elastography region of interest (ROI) was positioned within the renal cortex while carefully avoiding major vessels, collecting system structures, and focal lesions. Cortical measurements were obtained with clear corticomedullary differentiation whenever feasible. SWE measurements were acquired from the upper, middle, and lower poles of both kidneys. Three valid measurements were obtained from each region to account for the diffuse nature of renal involvement in CKD, and SWE values were recorded in kilopascals (kPa). The mean SWE value of both kidneys was calculated and used for statistical analysis. All examinations were performed by a radiologist experienced in abdominal ultrasonography and elastography.

Statistical analysis

All collected data were entered into an MS Excel spreadsheet and analyzed using Epi Info version 7.2.1.0 statistical software. Categorical variables were expressed as frequencies and percentages and analyzed using the Chi-square test. Continuous variables were presented as mean ± standard deviation (SD) and analyzed using the independent sample t-test or analysis of variance (ANOVA), as appropriate. The Pearson correlation coefficient was used to assess correlations between quantitative variables. Receiver operating characteristic (ROC) curve analysis was performed to determine the optimal cutoff value of SWE and to calculate sensitivity, specificity, positive predictive value, and negative predictive value. A p-value ≤ 0.05 was considered statistically significant.

## Results

The mean age of participants was comparable between the CKD and non-CKD groups (59.19 ± 7.95 years vs. 58.80 ± 8.58 years), with no statistically significant difference (p = 0.775). Similarly, gender distribution was comparable between the two groups, with males constituting 49 (65.3%) patients in the CKD group and 45 (60.0%) patients in the non-CKD group (p = 0.613). BMI was also similar in both groups (24.36 ± 3.03 vs. 23.58 ± 3.21 kg/m²; p = 0.130). These findings indicate that both groups were well matched in terms of baseline demographic characteristics (Table 1).

**Table 1:** Comparison of baseline characteristics and renal parameters between the study groups CKD: chronic kidney disease; CI: confidence interval; SD: standard deviation; BMI: body mass index; eGFR: estimated glomerular filtration rate; ACR: albumin-creatinine ratio; RRI: renal resistive index; SWE: shear wave elastography

Parameter	CKD (n = 75)	Test value	95% CI	Non-CKD (n = 75)	P-value
Age, years, mean ± SD	59.19 ± 7.95	t = 0.289	-2.28 to 3.06	58.80 ± 8.58	0.775
Male, n (%)	49 (65.3%)	χ² = 0.256	NA	45 (60.0%)	0.613
Female, n (%)	26 (34.7%)			30 (40%)	
BMI, kg/m², mean ± SD	24.36 ± 3.03	t = 1.530	-0.23 to 1.79	23.58 ± 3.21	0.130
eGFR, ml/min/1.73m², mean ± SD	59.92 ± 36.08	t = -11.681	-60.33 to -42.87	111.52 ± 12.72	< 0.001
Serum creatinine, mg/dl, mean ± SD	2.63 ± 1.37	t = 7.611	0.94 to 1.60	1.36 ± 0.46	< 0.001
ACR, mg/g, mean ± SD	201.17 ± 214.51	t = 7.650	140.62 to 238.58	11.57 ± 7.57	< 0.001
RRI, mean ± SD	0.69 ± 0.07	t = 12.771	0.152 to 0.208	0.51 ± 0.10	< 0.001
SWE, kPa, mean ± SD	11.19 ± 2.82	t = 8.493	2.21 to 3.55	8.31 ± 0.82	< 0.001

Renal function parameters showed significant differences between the two groups. Patients with CKD had significantly lower eGFR values compared to the non-CKD group (59.92 ± 36.08 vs. 111.52 ± 12.72 ml/min/1.73 m²; p < 0.001), indicating impaired renal function. Serum creatinine and ACR were significantly higher in the CKD group (2.63 ± 1.37 mg/dl and 201.17 ± 214.51 mg/g, respectively) compared to the non-CKD group (1.36 ± 0.46 mg/dl and 11.57 ± 7.57 mg/g, respectively; p < 0.001), reflecting worsening renal damage and increased proteinuria (Table 1).

RRI and SWE values were also significantly higher among CKD patients. The CKD group demonstrated a mean RRI of 0.69 ± 0.07 compared to 0.51 ± 0.10 in the non-CKD group (p < 0.001). Similarly, mean SWE values were significantly elevated in CKD patients (11.19 ± 2.82 kPa) compared to non-CKD patients (8.31 ± 0.82 kPa; p < 0.001). These findings suggest increased intrarenal vascular resistance and renal parenchymal stiffness in diabetic patients with CKD (Table 1).

Among the 75 patients with CKD, 20 patients (26.7%) in each group were classified as CKD Stage 1 and Stage 2. CKD Stage 4 comprised 15 patients (20.0%), whereas CKD Stage 3 and CKD Stage 5 each included 10 patients (13.3%). These findings indicate that the majority of patients were in the early to moderate stages of CKD (Table 2).

**Table 2:** Distribution of CKD patients by stage CKD: chronic kidney disease

CKD Stage	N (%)
Stage 1	20 (26.7)
Stage 2	20 (26.7)
Stage 3	10 (13.3)
Stage 4	15 (20)
Stage 5	10 (13.3)
Total	75 (100)

**Figure 1:**
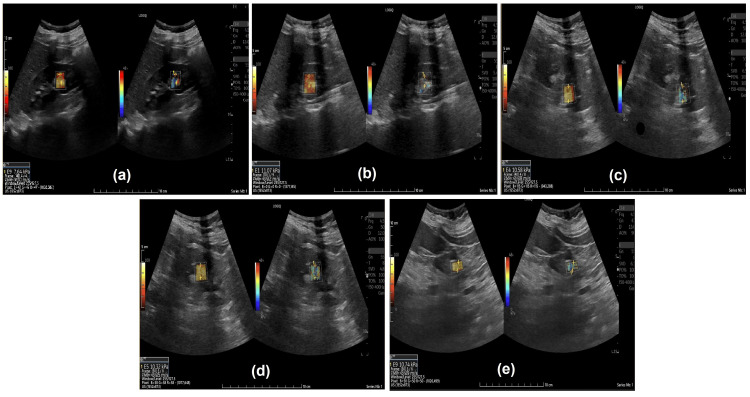
Representative renal SWE images demonstrating renal cortical stiffness measurements across CKD stages 1 to 5 in diabetic patients (a) 42-year-old diabetic female with Stage I CKD; (b) 64-year-old diabetic female with Stage 2 CKD; (c) 73-year-old diabetic male with Stage 3 CKD; (d) 67-year-old diabetic female with Stage 4 CKD; and (e) 45-year-old diabetic male with Stage 5 CKD SWE: shear wave elastography; CKD: chronic kidney disease

Representative SWE images illustrating renal cortical stiffness measurements across CKD stages 1 to 5 are shown in Figure 1. An overall increase in renal cortical stiffness was observed with advancing CKD stage.

A progressive decline in renal function was observed with advancing stages of CKD. Mean eGFR decreased significantly from 111.52 ± 12.72 ml/min/1.73 m² in the non-CKD group to 104.45 ± 6.91 ml/min/1.73 m² in CKD Stage 1 and further to 8.60 ± 2.99 ml/min/1.73 m² in CKD Stage 5 (p < 0.001). In contrast, serum creatinine levels increased progressively from 1.36 ± 0.46 mg/dl in the non-CKD group to 4.66 ± 1.47 mg/dl in CKD Stage 5 (p < 0.001), indicating worsening renal function with increasing CKD severity (Table 3).

**Table 3:** Comparison of renal parameters by CKD stage CKD: chronic kidney disease; SD: standard deviation; eGFR: estimated glomerular filtration rate; ACR: albumin-creatinine ratio; RRI: renal resistive index; SWE: shear wave elastography

Parameter	Non-CKD, mean ± SD	CKD 1, mean ± SD	CKD 2, mean ± SD	CKD 3, mean ± SD	CKD 4, mean ± SD	CKD 5, mean ± SD	F value	P-value
eGFR (ml/min/1.73m²)	111.52 ± 12.72	104.45 ± 6.91	75.75 ± 7.88	45 ± 9.64	23.6 ± 4.69	8.6 ± 2.99	363.40	< 0.001
Serum creatinine (mg/dl)	1.36 ± 0.46	1.59 ± 0.26	1.86 ± 0.30	2.26 ± 0.69	3.94 ± 0.86	4.66 ± 1.47	88.66	< 0.001
ACR (mg/g)	11.87 ± 7.57	13.45 ± 9.38	63.5 ± 22.03	242 ± 121.55	349.87 ± 84.35	588.1 ± 160.6	256.47	< 0.001
RRI	0.51 ± 0.10	0.64 ± 0.05	0.64 ± 0.03	0.69 ± 0.03	0.74 ± 0.03	0.82 ± 0.05	51.99	< 0.001
SWE (kPa)	8.31 ± 0.82	8.88 ± 0.62	10.6 ± 1.13	10.7 ± 1.00	11.93 ± 1.83	16.39 ± 3.45	83.26	< 0.001

ACR also demonstrated a marked stepwise increase across CKD stages, rising from 11.87 ± 7.57 mg/g in the non-CKD group to 588.10 ± 160.60 mg/g in CKD Stage 5 (p < 0.001), reflecting progressive proteinuria and glomerular damage (Table 3). Similarly, RRI values increased from 0.51 ± 0.10 in the non-CKD group to 0.82 ± 0.05 in CKD Stage 5 (p < 0.001), indicating increasing intrarenal vascular resistance. SWE values also showed a significant stepwise rise from 8.31 ± 0.82 kPa to 16.39 ± 3.45 kPa (p < 0.001), suggesting progressive renal parenchymal stiffness and fibrosis with advancing CKD stages (Table 3).

Correlation analysis showed a strong negative correlation between RRI and eGFR (r = -0.722, p < 0.001) and a strong positive correlation between RRI and ACR (r = 0.657, p < 0.001), indicating that higher intrarenal vascular resistance was associated with worsening renal function and greater albuminuria. Similarly, SWE demonstrated a strong negative correlation with eGFR (r = -0.777, p < 0.001) and a strong positive correlation with ACR (r = 0.791, p < 0.001), suggesting that increasing renal stiffness was associated with declining renal function and increased proteinuria. A significant positive correlation was also observed between SWE and RRI (r = 0.617, p < 0.001), indicating an association between increasing renal parenchymal stiffness and higher intrarenal vascular resistance in patients with CKD (Table 4).

**Table 4:** Correlation of RRI and SWE with renal parameters RRI: renal resistive index; SWE: shear wave elastography; eGFR: estimated glomerular filtration rate; ACR: albumin-creatinine ratio

Variables	Correlation coefficient (r)	P-value
RRI vs. eGFR	-0.722	< 0.001
RRI vs. ACR	0.657	< 0.001
SWE vs. eGFR	-0.777	< 0.001
SWE vs. ACR	0.791	< 0.001
SWE vs. RRI	0.617	< 0.001

ROC analysis showed that both RRI and SWE were effective predictors of CKD. RRI demonstrated excellent diagnostic accuracy with an AUC of 0.924 (95% CI: 0.884-0.964; p < 0.001). At a cutoff value of 0.575, RRI showed a sensitivity of 72% and specificity of 100%, indicating excellent ability to correctly identify patients without CKD. Similarly, SWE demonstrated high diagnostic accuracy with an AUC of 0.899 (95% CI: 0.850-0.948; p < 0.001). A cutoff value of 9.65 kPa showed 100% sensitivity and 68% specificity, indicating excellent ability of SWE to detect CKD cases, although with comparatively lower specificity than RRI (Table 5).

**Table 5:** ROC analysis of RRI and SWE for the prediction of CKD ROC: receiver operating characteristic; RRI: renal resistive index; SWE: shear wave elastography; CKD: chronic kidney disease; AUC: area under the curve; CI: confidence interval

Parameter	AUC (95% CI)	Cutoff value	Sensitivity	Specificity	P-value
RRI	0.924 (0.884–0.964)	0.575	72%	100%	< 0.001
SWE	0.899 (0.850–0.948)	9.65 kPa	100%	68%	< 0.001

## Discussion

The present study was conducted to evaluate the role of RRI and SWE in differentiating diabetic patients with CKD from diabetic patients without CKD and to assess their relationship with conventional renal function parameters. DKD is one of the most important complications of diabetes mellitus and is often diagnosed only after significant structural and functional renal damage has occurred. Conventional biochemical markers such as serum creatinine and eGFR mainly reflect functional impairment, whereas imaging techniques such as SWE may help detect earlier structural changes related to fibrosis and renal remodeling.

In the present study, both groups were comparable with respect to age, gender, and BMI, indicating that baseline demographic variables were well matched. The majority of patients belonged to the fifth and sixth decades of life, which is similar to studies by Yu et al. [11], Goya et al. [8], and Bob et al. [3], where middle-aged diabetic populations were predominantly affected. Male predominance observed in the present study was also consistent with findings reported by Hassan et al. [2], Rehman et al. [12], and Shaker et al. [13]. The absence of significant differences in demographic parameters reduces the possibility of confounding influences on renal stiffness and Doppler measurements.

Renal function parameters showed significant differences between diabetic patients with CKD and those without CKD. Patients with CKD demonstrated significantly lower eGFR and significantly higher serum creatinine and ACR values. Furthermore, these parameters worsened progressively with advancing CKD stages. Similar findings have been reported by Samir et al. [1], Yu et al. [11], and Goya et al. [8], who also observed progressive biochemical deterioration with worsening CKD. These findings confirm appropriate staging of CKD in the present study population and provide a reliable basis for comparison with imaging parameters.

The present study demonstrated significantly higher RRI values in diabetic patients with CKD compared to those without CKD. In addition, RRI increased progressively with advancing CKD stages. Increased RRI reflects elevated intrarenal vascular resistance resulting from glomerulosclerosis, vascular narrowing, and interstitial fibrosis. Similar observations were reported by Rehman et al. [12], who found a significant inverse relationship between RRI and eGFR. Kırteke et al. [14] also reported elevated resistive index values in diabetic patients even before overt CKD became clinically evident. These findings suggest that RRI may be a useful marker for identifying renal vascular changes in diabetic nephropathy. However, RRI may also be influenced by systemic factors such as blood pressure, vascular compliance, and heart rate, which may affect its specificity.

The most important findings of the present study were related to SWE. Mean SWE values were significantly higher in diabetic patients with CKD compared to those without CKD, and SWE values increased progressively with advancing CKD stages. These findings suggest increasing renal parenchymal stiffness with worsening renal disease. CKD is characterized by progressive fibrosis, tubular atrophy, and extracellular matrix deposition, all of which contribute to increased tissue stiffness. Similar progressive increases in renal stiffness have been reported by Yu et al. [11], Goya et al. [8], Bob et al. [3], Hassan et al. [2], Koc et al. [15], and Shi et al. [16]. These studies also demonstrated that SWE abnormalities may be present even during early stages of diabetic nephropathy before major structural changes become apparent on conventional ultrasonography.

Correlation analysis in the present study showed a strong negative correlation between SWE and eGFR, indicating that increasing renal stiffness is associated with declining renal function. A strong positive correlation was also observed between SWE and ACR, suggesting that higher renal stiffness is associated with worsening proteinuria. Similar findings have been reported by Yu et al. [11], Goya et al. [8], and Koc et al. [15]. Histopathology-based studies by Shi et al. [16] and Radulescu et al. [17] have further strengthened these observations by demonstrating a close relationship between SWE values and renal fibrosis severity.

A significant positive correlation between SWE and RRI was also observed in the present study. This finding indicates that increasing renal stiffness is associated with increasing intrarenal vascular resistance. Similar associations have been described by Kırteke et al. and Rehman et al. [12], who suggested that combined assessment of SWE and Doppler indices may improve diagnostic evaluation in CKD. Pathophysiologically, progressive fibrosis can compress intrarenal vessels, leading to increased vascular resistance and reduced perfusion, thereby explaining the simultaneous rise in both parameters.

ROC analysis further demonstrated the diagnostic utility of both imaging modalities. RRI showed excellent specificity, while SWE demonstrated excellent sensitivity for identifying CKD. Similar findings were reported by Samir et al. [1], who demonstrated good diagnostic accuracy of SWE in CKD patients. Lim et al. [18] also emphasized the value of combining elastography and Doppler assessment for improved diagnostic confidence and assessment of renal involvement.

Strengths and limitations

The present study directly compared diabetic patients with and without CKD, enabling better evaluation of SWE and RRI in DKD. Multiple renal parameters, including eGFR, serum creatinine, ACR, RRI, and SWE, were assessed, providing a comprehensive renal evaluation. Standardized SWE techniques and multiple cortical measurements improved the reliability of results. However, the study had certain limitations. It was a single-center study with a relatively small sample size, which may limit generalizability. Renal biopsy correlation was not performed due to the invasive nature of the procedure. SWE measurements may also be affected by technical and patient-related factors such as hydration status, respiratory motion, and operator dependency. Respiratory motion, breath-holding level, inspiratory depth, and transducer pressure may influence SWE measurements despite attempts at standardization, as previously highlighted in abdominal elastography studies by Kaya et al. [19].

Furthermore, interobserver and intraobserver variability assessments were not performed, which may affect the reproducibility and generalizability of SWE measurements. Detailed gray-scale ultrasonographic findings such as cortical echogenicity, corticomedullary differentiation, cortical thickness, and renal size were not systematically analyzed and correlated with SWE measurements. Additionally, the cross-sectional design did not allow assessment of longitudinal changes, predictive performance, or long-term clinical outcomes.

## Conclusions

The present study demonstrated that diabetic patients with CKD had significantly higher RRI and SWE values compared to diabetic patients without CKD. Both parameters showed a progressive increase with advancing CKD stages and demonstrated significant correlation with conventional renal function markers such as eGFR and ACR. SWE showed high sensitivity, while RRI demonstrated high specificity for identifying CKD in the study population. These findings suggest that SWE and RRI may serve as useful non-invasive imaging biomarkers for assessment and risk stratification in DKD. Further prospective longitudinal studies are required to validate their predictive and monitoring utility.
